# Personalized Medicine Approach in Treating Parkinson’s Disease, Using Oral Administration of Levodopa/Carbidopa Microtablets in Clinical Practice

**DOI:** 10.3390/jpm11080720

**Published:** 2021-07-26

**Authors:** Helga María Grétarsdóttir, Erik Widman, Anders Johansson, Dag Nyholm

**Affiliations:** 1 Department of Neuroscience, Neurology, Uppsala University, 75185 Uppsala, Sweden; helga.maria.gretarsdottir@akademiska.se (H.M.G.); erikwidman1@hotmail.com (E.W.); 2 Department of Clinical Neuroscience, Karolinska Institutet, 17177 Stockholm, Sweden; anders.johansson@ki.se

**Keywords:** Parkinson’s disease, levodopa, carbidopa, microtablets, dose dispenser

## Abstract

Background: The most effective symptomatic treatment in Parkinson’s disease (PD) is levodopa in standard doses. However, as the disease progresses, there may be a need for a more personalized approach and fine tuning, in accordance with the patients’ needs. This study aims to evaluate the individual experience of levodopa/carbidopa 5/1.25 mg microtablets (LC-5) in clinical practice with respect to efficacy, tolerability, and usability. The method used was as follows: patients answered a questionnaire concerning the effect and usability of LC-5, and their medical records were reviewed. Regarding results, thirty-five survey responses were obtained, and 29 patients’ medical records were reviewed. The LC-5 dose dispenser usability was generally rated positively and facilitated medication adherence. The majority (85%) of patients reported symptom improvement while using LC-5, compared with previous standard treatments. These results suggest that LC-5 therapy is generally well-tolerated, with favorable patient-reported efficacy and user friendliness, as well as the possibility for an individualized, fine-tuned PD treatment. Further studies with a prospective design and larger study population are needed to confirm the results.

## 1. Introduction

Personalized medicine, widely conflated with precision medicine, is a medical model which regards each patient’s specific biological profile and needs when guiding decisions concerning the treatment of a disease [1]. Combining individual data and clinical phenotypes allows for the advancement of better targeted therapies, by selecting the optimal treatment for each patient [2]. This is important when considering multifactorial pathology-driven conditions, which may require the use of “cocktail therapies”, particularly relevant in Parkinson’s disease (PD) with complex combinations of motor and nonmotor symptoms [3,4].

Symptomatic PD treatment targets the dopaminergic deficit due to the degeneration of neurons in the substantia nigra [2]. Since the introduction of levodopa in the 1960s, it has been the gold standard for PD treatment [5]. However, as the disease progresses, the pharmacological response may vary, requiring higher and more frequent doses of levodopa [2,6,7,8]. Patients may develop motor fluctuations over time, manifested as end-of-dose deterioration and/or dyskinesia, affecting nearly half of PD patients after five years of treatment [2,9,10].

By implementing the personalized medicine model for PD, the levodopa dose would be fractionated to smaller doses, taken at the onset of motor fluctuations and allowing for constant modification due to the dynamic nature of the disease [11,12]. One such possibility is the use of levodopa/carbidopa 5/1.25 mg microtablets (LC-5) in an electronic dose dispenser [13], allowing the individualization and fine tuning of the dose size and interval, as well as a more stable levodopa plasma concentration, compared with standard tablet formulations [7,8,14,15,16]. The dose dispenser is equipped with an alarm, to facilitate treatment adherence, and an optional diary-like function for self-reporting [9]. The aim of this study was to assess the individual LC-5 experience in clinical practice with respect to efficacy, tolerability, and usability.

## 2. Materials and Methods

This was a retrospective, observational study of PD patients previously or currently treated with LC-5 microtablets at seven sites in Sweden.

The LC-5 is a dispersible microtablet formulation for oral intake, containing 5/1.25 mg of levodopa/carbidopa (Flexilev^®^, Sensidose AB, Sollentuna, Sweden). The microtablets are delivered in cartridges with 750 tablets in each. The cartridge is placed inside an electronic dose dispenser (My Flexible Individual Dosing, MyFID^®^, Sensidose AB, Sollentuna, Sweden). The dispenser is operated via a touch screen, where the daily dosage is pre-programmed by the physician in an individualized fashion, in terms of daily levodopa dose and the number of dose intakes. Each dose size can be tailored in steps of 5 mg levodopa and dose intakes can be adjusted in 5-minute intervals. The patient is alerted for dose intake by the built-in alarm and is usually given a range for fine-tuning each dose. The dose is released from the dispenser, preferably into a glass of water, and the microtablets are immediately dispersed.

LC-5 was approved in Sweden in 2014 and in the European Union in 2016. In Sweden, LC-5 is reimbursed only for patients who would otherwise be treated with apomorphine or levodopa infusions.

In total, 70 patients on LC-5 were identified at seven study sites. A total of 35 patients filled out a questionnaire, 5 of which did not sign a consent form but mailed in their questionnaire anonymously and are, thus, included in the analyses. The questionnaire that the patients answered (taken from a previous survey 2017) concerned their general experience of efficacy, as well as the activity of daily living and the usability of the dose dispenser (buttons, navigation through the menus, tablet dispensing, cartridge replacement, and general portability). Patients also provided information regarding frequency, duration, and severity of motor function (bradykinesia, troublesome dyskinesia, and non-troublesome dyskinesia).

A review of medical records was conducted until 2020 and data retrospectively collected with respect to age, sex, PD duration, treatment duration, medication/dosage before and during LC-5 treatment, side effects, and technical problems. The patients initiated LC-5 treatment due to unsatisfactory effects or difficulty finding the right dose with previous PD medications, as well as motor fluctuations and/or dyskinesia. One patient initiated LC-5 due to severe neurogenic orthostatic hypotension, which worsened on standard formulations of levodopa.

Medical records of 29 patients were reviewed (of the 35, 5 did not sign the consent form and 1 continued treatment in another city). One patient died of causes unrelated to LC-5 prior to filling out the survey, however the medical records were obtained.

The study was approved by the Regional Ethics Committee and written consent forms were signed by the 30 patients, as described above. Only descriptive data is reported, mainly as median and range due to non-normal distribution of the dataset, and statistical analyses were considered irrelevant in this small, open, observational report.

## 3. Results

### 3.1. Patient Records

The 29 patients had a median PD duration of 13 years; the median age was 70 years and 62% were females (Table 1). The total number of days treated with LC-5 varied between patients, with a median (range) of 810 (131–2542) days. Six patients (21%) discontinued the LC-5 treatment after 131–688 days due to general progression of the disease and the implementation of a more invasive therapy.

Five patients reported a LC-5 device complication: four patients had to replace their devices due to breakage and one reported a faulty number of tablets dispensed.

Two patients reported side effects that they attributed to the LC-5 treatment. One patient reported dizziness and nightmares, while the other reported a rash and nausea. Neither required medication discontinuation.

### 3.2. Treatments

Before the initiation of LC-5, 28 patients used a L-DOPA/decarboxylase inhibitor, while 23 patients were treated with dopamine agonists (pramipexole = 11, ropinirole = 6, and rotigotine = 6) (Table 2).

Dopamine agonists were also used (*n* = 18) after initiating the LC-5 treatment (pramipexole = 10, ropinirole = 4, and rotigotine = 4), while the use of additional levodopa decreased to 10 (mainly nighttime sustained-release formulations). The median number of dopaminergic medications per patient was 3, both before and after LC-5 treatment. The median (range) daily dose of levodopa before LC-5 was 600 (0–1925) mg, and the levodopa-equivalent dose (LED) was calculated according to [17] 878 (25–2160) mg, compared to the last/latest daily dose of 690 (335–2210) mg and LED 910 (535–2286) mg. The reason for one patient to start with LC-5 after 0 mg of levodopa at baseline was severe neurogenic orthostatic hypotension.

Four patients used LC-5 as concomitant therapy to bilateral subthalamic deep brain stimulation.

The median (range) number of daily LC-5 doses at the last/latest follow-up was 8 (4–17), and the morning dose was 110 (40–300) mg. The median dose interval was 120 min, ranging from 60 min in 4 patients to 240 min in two patients.

### 3.3. Survey

The results of the survey are presented in Table 3. Before the LC-5 dispenser treatment, 18 of 34 patients used an aid (alarm, pill box) to remind them to take their medication.

**Figure 1:**
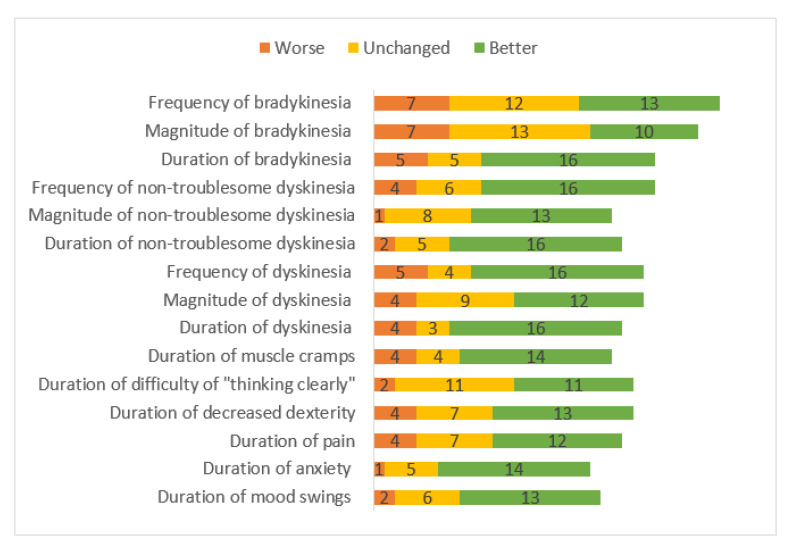
The patient-reported experienced change in bradykinesia and dyskinesia, as well as mood swings, pain, cognitive symptoms, and muscle cramps during LC-5 treatment. The response alternatives for “much worse” and “worse” are combined as “worse”, as well as “better” and “much better”, combined as “better”.

Patients reported taking LC-5 doses, on average, 7.5 times, compared to 5 times while on their previous medication; however, they perceived this increased frequency as an improvement, compared to the previous situation (24 better, 4 neutral, and 1 worse). Twenty-two LC-5 patients reported taking extra doses when needed, whereas 9 did not. Most of the patients experienced improvement regarding dyskinesia duration, magnitude, and frequency (Figure 1). The patients also reported improvement in non-motor symptoms, including shorter duration of mood swings, anxiety, bradyphrenia, general pain, and muscle cramps.

#### Practical Handling of the Dose Dispenser

Most patients reported user-friendliness when it came to the dose dispenser device (Table 3). Six patients had some difficulty bringing the dose dispenser with them in everyday life, potentially due to its size (large/too large, *n* = 14), weight (heavy, *n* = 7), or the need to dissolve the tablets with water. Five patients found the device to be small/too small, seven patients reported it to be too light, and 19 patients were neutral in terms of weight. One patient had produced a wooden stand for the dose dispenser. Five patients spontaneously stated that contact with a support nurse was important and helpful, in case of technical problems.

## 4. Discussion

Advanced PD with declining motor function requires individualized treatment with the possibility to adapt to daily circumstances (the concept of dose fractionation), in other words, that slightly smaller doses can be administered more often, based on patients’ needs [6,11]. This is a common strategy during the course of PD, prompted by the development of wearing-off and dyskinesia [6]. The dose fractioning concept was the reason for the patients’ initiation of LC-5 treatment. In the current study, data from the patients’ records indicated a greater diversity of daily levodopa dose sizes and dosing intervals with LC-5, as compared with previous therapy.

The strategy of continuous drug delivery is also successful in severely fluctuating patients, as shown by studies on apomorphine and levodopa infusions [18,19,20]. Levodopa fractionation could thus be beneficial already before the motor complications occur; for example, 6 doses per day, rather than the standard 3–4. Although, the level of evidence is presently not robust [21].

In the present study, most of the patients (85%) experienced self-rated treatment improvement with LC-5. The preponderance of the patients found the duration, magnitude, and the frequency of their dyskinesia and non-troublesome dyskinesia to be improved, compared to previous treatments. This improvement is possibly explained by the medication fine-tuning option of the LC-5. Some patients reported a shorter duration of bradykinesia, but its magnitude and frequency were perceived as more or less unchanged. There was an improvement in the non-motor symptoms with LC-5 usage, including the duration, magnitude, and frequency of pain, anxiety, and mood swings (*n* = 13), though not the frequency of the mood swings. LC-5 treatment did not affect the ability to think clearly, nor the frequency of decreased dexterity.

Most patients found the dose dispenser to be user-friendly, however a few patients (27%) found it difficult to change the cartridge, and a limited number of patients (35%) used the diary function (registering symptoms into the device), both aspects which may be improved with increased patient education in handling the dispenser and/or improved technical design. A few patients found it difficult to travel with the dose dispenser due to the size and weight of the dispenser or the need to dissolve the tablets with water, which could be resolved with the production of sub-lingual dissolving tablets. In fact, the microtablets dissolve rapidly in water or saliva, and six patients reported ingesting the tablets whole, without dissolving them in water. Some patients bring plastic tubes containing planned doses when they leave home, so that they do not have to carry the dose dispenser. Despite the potential difficulties with a technical device, most patients (88%) found the device (as an equipment to facilitate treatment) easier, compared to previous therapies. Additionally, most patients (81%) found it easier to remember to take their medication due to the LC-5 alarm feature, thus increasing adherence.

The present study’s major limitation was the low response rate, where 50% of the eligible patients answered the survey and a written consent for analyzing medical records was obtained from 43%. There is a possibility that the survey responders were more pleased with the LC-5 treatment, implying a selection bias. This study was based on the patients recall with a possible recall bias. PD patients with cognitive decline/dementia might have difficulties answering the survey, and thus, the results are not generalizable to all PD patients using LC-5. Further, the retrospective, uncontrolled, and unblinded design is a clear limitation for drawing evidence-based conclusions. Prospective clinical trials, using validated rating scales for motor and non-motor assessments, are necessary.

Future research and treatment could also focus on using sensor-based algorithms for improved medication tailored to fit the needs of each patient [22]. In addition, better use of the diary/registration function could help the health personnel and patients adjust the medication for the optimal treatment response. Thomas et al. showed it to be feasible and effective to use algorithmic, sensor-based dosing adjustments to optimize treatment, which was best suited for each patients needs [22]. This was conducted by having the supervising neurologist make dosing adjustments based on data from the Parkinson’s KinetiGraph™ (PKG) that the patients wore for a week during their everyday life [22].

In the present study, women were over-represented (62%), which is uncommon in studies of advanced PD treatments [23,24]. There may be many different explanations for this gender imbalance, but it cannot be ruled out that women, due to lower body weight, are more sensitive to small changes in levodopa doses, and thus, on a group level, are more prone to benefit from levodopa fractionation [25,26,27]. Another subgroup of PD patients, where small dose increments are required, are those with severe orthostatic hypotension; the same situation may occur in patients with nausea, or other adverse effects, from levodopa. In the present study, LC-5 was combined with DBS in four cases. This is an interesting combination of the two fine-tuned treatments, and a less expensive alternative than combining DBS and levodopa infusions [28].

In conclusion, this is the largest report on the clinical use of LC-5 to date. It shows that LC-5 therapy is generally well-tolerated, with favorable patient-reported efficacy and user friendliness, as well as a suitable part of the personalized medicine model of individualizing treatment for each patient. Moreover, it is possible to use LC-5 as a long-term therapy for PD. Prospective, double-blind studies are required for improving the evidence-level of LC-5.

## Figures and Tables

**Table 1:** Demographics based on data collected from medical records (*n* = 29).

Variable	Statistics
Age in years, median (range)	70 (54–82)
Gender (male: female)	11:18
PD duration, years, median (range)	13 (3–31)
LC-5 duration, days/years, median (range)	810 (131–2542) days/2.2 (0.4–7.0) years
Device complication (*n*)	5 (17%)

**Table 2:** Treatment with other dopaminergic Parkinson’s disease (PD) medication prior to LC-5 and concomitant PD treatment during LC-5 therapy (*n* = 29).

PD Medication	Number of Patients, Prior to LC-5	Number of Patients, during LC-5
Levodopa/decarboxylase inhibitor	22	12
Entacapone; Levodopa/carbidopa/entacapone	6	4
Amantadine	3	0
Dopamine agonist	23	18
MAO-B-I	14	14
Median daily dose of L-DOPA (mg)	600	690
Median LED (mg)	878	910

One patient was not treated with levodopa prior to LC-5 due to severe neurogenic orthostatic hypotension, which worsened with previous attempts using standard levodopa formulations. LC-5: levodopa-carbidopa microtablets; LED: levodopa-equivalent daily dose; MAO-B-I: monoamine oxidase B inhibitor.

**Table 3:** Questions asked with answers (*n* = 33 if not otherwise stated).

Questions	Improved	Unchanged	Worsened	Don’t Know
Experienced effect of LC-5 on disease symptoms compared with previous treatment? ^a^	28	5	1	-
How do you feel it is possible to adjust the dose size with the MyFID dose and the micro-tablets compared to your usual treatment? ^b^	20	10	-	-
Does/did the dose dispenser facilitate remembering to take your tablets? ^c^	28	1	3	-
Does/did the dose dispenser simplify or complicate your treatment in general? ^a^	30	2	2	-
	**Well**		**Not well**	**Don’t know**
What is/was your experience in seeing the screen?	31		2	-
What is/was your experience in pressing the buttons?	32		1	-
What is/was your experience in dispensing the tablets?	31		2	-
What is/was your experience in entering symptoms? ^d^	8		2	17
What is/was your experience in changing the cartridge?	24		9	-
What is/was your experience regarding the portability of the dose dispenser in your everyday life? ^e^	24		7	-

^a^*n* = 34, ^b^ *n* = 30, ^c^ *n* = 32, ^d^ *n* = 27, ^e^ *n* = 31.

## Data Availability

The data presented in this study are available on request from the corresponding author. The data are not publicly available due to privacy.
